# Sinensetin ameliorates established high-fat diet-induced liver injury and intestinal barrier dysfunction through the mitophagy/TLR4/MAPK signaling pathway

**DOI:** 10.3389/fnut.2026.1812651

**Published:** 2026-06-24

**Authors:** Zhuoqun Meng, Qin Zhang, Zhichang Zhao, Yuan Zhang, Zhijie Lu, Guirong Liu, Wenliang Xiang, Qing Zhang, Jie Tang

**Affiliations:** 1 School of Food and Bioengineering, Xihua University, Chengdu, Sichuan, China; 2 China Agricultural University-Sichuan Advanced Agricultural & Industrial Institute, Chengdu, China

**Keywords:** gut–liver axis, high-fat diet, intestinal barrier dysfunction, liver injury, mitophagy, sinensetin

## Abstract

**Introduction:**

Long-term consumption of a high-fat diet (HFD) causes liver injury characterized by steatosis, inflammation, and fibrosis. Mitophagy, as a selective autophagy, is reported to be involved in the regulation of liver injury. Sinensetin, a polymethoxylated flavonoid abundant in citrus fruit peels, exhibits various biological activities, including anti-inflammatory and hepatoprotective properties. However, whether sinensetin can target mitophagy and protect against HFD-induced liver damage via the gut–liver axis remains inadequately explored.

**Methods:**

In order to further investigate the relationships involved, we conducted histopathology analysis, biochemical analysis, 16S rRNA sequencing, and short-chain fatty acid (SCFA) levels.

**Results:**

Sinensetin administration ameliorated hepatic steatosis, inflammation, and restored intestinal integrity in HFD-fed mice. Mechanistically, sinensetin remodeled the gut microbiota, elevating SCFA levels, which activated mitophagy and cleared damaged mitochondria in liver and intestinal tissues, thereby suppressing the toll-like receptor 4 (TLR4)/mitogen-activated protein kinase (MAPK) signaling.

**Conclusion:**

Sinensetin may contribute to protecting against HFD-induced liver injury and intestinal barrier dysfunction by orchestrating the integrated “microbiota-SCFA-mitophagy” defensive network, providing a novel paradigm beyond the classical TLR4/MAPK axis.

## 1 Introduction

A high-fat diet (HFD), a key driver of metabolic disorders and obesity, poses a major global health concern due to its strong association with cardiometabolic diseases such as type 2 diabetes, heart disease, and non-alcoholic fatty liver disease (NAFLD) (1). Of particular concern is its rising prevalence among younger populations, foretelling persistent health issues and significant socioeconomic burdens (2). The liver injury caused by HFD is not an isolated event, but is closely related to intestinal microbiota disorders, intestinal mucosal barrier injury, and enterogenous endotoxemia, in which the “gut liver axis” plays a central role (3). Moreover, dietary bioactive lipids like conjugated linoleic acid (CLA) and omega-3 fatty acids (DHA, EPA) reduce oxidative stress and apoptosis contributing to the inhibition of ischemia/reperfusion induced renal injury partly via modulation of mTOR mediated signaling pathways (4, 5). For instance, through a process initiated by increased reactive oxygen species (ROS) generation from the gut microbiota, an HFD disrupts the intestinal barrier, ultimately culminating in bacterial translocation and liver inflammation (6). Additionally, HFD reshapes microbial communities, reducing beneficial taxa (e.g., *Bacteroidetes* and *Lactobacillus*) while promoting pathogenic bacteria, such as *Enterobacteriaceae*, which further aggravates liver injury by modulating bile acid metabolism and short-chain fatty acid (SCFA) synthesis (7). Therefore, in-depth exploration of the core mechanism of the “gut liver axis” is of great scientific significance for a comprehensive understanding and prevention of HFD related liver injury.

When the gut–liver axis is disrupted, it compromises hepatic health, contributing to the pathogenesis of NAFLD by upregulating pro-inflammatory signals like toll-like receptor 4 (TLR4) and NOD-like receptor family pyrin domain containing 3 (NLRP3) (8). This increased intestinal permeability allows a significant amount of LPS from the gut to enter the liver via the portal vein. LPS binds to the TLR4 receptors, triggering downstream signaling cascades involving mitogen-activated protein kinase (MAPK) and nuclear factor-κB (NF-κB). This ultimately leads to the massive release of pro-inflammatory cytokines such as tumor necrosis factor-alpha (TNF-α) and interleukin-6 (IL-6), driving hepatic inflammation, steatosis, and even fibrosis (9). Various complex signaling pathways could be stimulated by activated TLR4, such as the activation of MAPKs, myeloid differentiation factor 88 (MyD88)-independent and NF-κB pathway (10, 11). Research indicates that inhibiting the TLR4/NF-κB/MAPK signaling pathway can mitigate liver injury induced by an HFD (12, 13). However, classical inflammatory cascades like TLR4/MAPK are downstream manifestations of metabolic imbalance. Recent perspectives emphasize that gut microbiota-derived metabolites, particularly SCFAs, act as crucial upstream regulators that not only suppress these inflammatory signals but also govern intracellular organelle homeostasis (14).

Emerging evidence underscores the crucial role of mitophagy, a selective form of autophagy for mitochondrial quality control, in maintaining liver homeostasis (15). The core mechanism involves the stabilization of PTEN-induced putative kinase 1 (PINK1), a serine/threonine kinase, on the outer membrane of damaged mitochondria. This stabilization promotes the phosphorylation and subsequent activation of the cytosolic E3 ubiquitin ligase Parkin, which is then recruited to initiate the autophagic removal of impaired organelles (16, 17). Activated TLR4/MAPK signaling can inhibit mitophagy activity, suppressing the clearance of damaged mitochondria. Conversely, defective mitophagy leads to the accumulation of dysfunctional mitochondria, which release reactive oxygen species (mtROS) and mitochondrial DNA (mtDNA) (16). These molecules act as potent activators of the TLR4 pathway and its downstream MAPK/NF-κB cascades, sustaining a cycle of chronic inflammation (18, 19). Crucially, an emerging paradigm highlights the profound crosstalk between gut microbiota-derived SCFAs and intracellular mitophagy. SCFAs not only maintain intestinal barrier integrity but also serve as potent signaling molecules to initiate PINK1/Parkin-mediated mitophagy, thereby forming a synergistic “microbiota-SCFA-mitophagy” defensive network.

Phytochemical-rich fruits have been demonstrated to offer diverse health benefits, having the potential to prevent various diseases, such as hepatic injury (20, 21). Sinensetin, a polymethoxylated flavonoid prevalent in citrus fruits, has a well-established safety profile for human consumption and exhibits a broad pharmacological profile that encompasses anti-inflammatory, antioxidant, and anti-cancer activities (22, 23). Sinensetin has been shown to protect against acute liver injury caused by acetaminophen (24) and NAFLD induced by an HFD (25). Sinensetin effectively modulates NF-κB and MAPK signaling pathway activity (22). Studies have shown that sinensetin can alleviate osteoarthritis and hepatocellular carcinoma by inducing autophagy (26, 27). However, whether sinensetin can alleviate HFD-induced liver damage by orchestrating the integration of gut microbiota remodeling, SCFA production, and mitophagy remains inadequately explored.

To investigate HFD-induced liver injury, we established a mouse model. Our findings revealed that sinensetin ameliorated this damage by modulating the integration of mitophagy, gut microbiota, and SCFAs, driving a paradigm shift beyond the classical TLR4/MAPK signaling. The results demonstrate sinensetin is associated with protecting liver function through mitophagy and gut–liver interactions, revealing novel therapeutic applications. Additionally, they provide a substantial scientific basis for the development and utilization of citrus peel by-products.

## 2 Materials and methods

### 2.1 Reagents and antibodies

The sinensetin standard (≥98.0%, purity) was purchased from Chengdu Yijierui Biotechnology Co., Ltd. (Chengdu, China). Anti-PINK1, anti-Parkin, anti-P62, anti-LC3, anti-p-JNK, anti-JNK, anti-p38, anti-TLR4, anti-ZO-1, anti-occludin, and anti-GAPDH were acquired from Affinity Biosciences Corp located in Jiangsu, China. Additionally, antibodies against p-ERK, ERK, and p-p38 were acquired from ZenBio Biosciences Corp, located in Chengdu, China.

### 2.2 Animals and treatment

All experimental procedures were conducted using male C57BL/6J mice, 6 to 8 weeks old, purchased from Sichuan Lilaisinuo Biological Technology Co., LTD. The animal experiment in this study was conducted in strict accordance with the National Institutes of Health (NIH) Guide for the Care and Use of Laboratory Animals. The animal experiment protocol was fully reviewed and approved by the Institutional Animal Care and Use Committee (IACUC) of Sichuan Lilaisinuo Biological Technology Co., LTD. (ethical license number: LLSN-2025138). Furthermore, the design and reporting of our animal experiments followed the (Animal Research: Reporting of *In Vivo* Experiments) ARRIVE guidelines to ensure the reliability and transparency of the *in vivo* data. The animals were housed under standard laboratory conditions maintained at 23 °C (±1 °C) with 55% (±10%) humidity and a regulated 12-h day/night cycle. After a week of acclimatization to a specific feeding regimen, mice were randomly divided into four groups: a normal control diet, an HFD, an HFD with 25 mg/kg sinensetin (HFD + sinensetin-L), and an HFD with 50 mg/kg sinensetin (HFD + sinensetin-H). Each group consisted of six mice. Mice in the HFD and HFD + sinensetin groups were fed a 60 kcal% HFD (D12492, Xiaoshuyoutai, Beijing, China) for 12 weeks to induce liver injury (28), while the control group received a standard diet. During week 13, sinensetin was daily administered via oral gavage, dissolved in the normal saline. Previous studies determined the dosage of sinensetin (29). During the treatment period, the control and HFD groups were given 0.1 mL of the normal saline through oral gavage. No mortality was observed in any of the experimental groups throughout the entire duration of the study. Daily monitoring of the animals’ general health, behavior, and body weight confirmed that the HFD and the oral administration of sinensetin were well-tolerated and did not induce acute toxicity or lethality. At the end of the 16-week period, mice were fasted for 12 h, then deeply anesthetized by intraperitoneal injection of 1% pentobarbital sodium. Mice were anaesthetized for orbital blood collection to facilitate blood sample collection. Tissue samples of the small intestine and liver were promptly gathered, snap-frozen, weighed, and kept at −80 °C for later analysis.

### 2.3 Histopathology analysis

Murine liver and small intestine tissues were fixed in 4% paraformaldehyde, dehydrated, and embedded in paraffin following standard protocols. Subsequently, 4 μm thick tissue sections were mounted on glass slides. Tissue sections of the liver and small intestine were stained with hematoxylin and eosin (H&E) and examined through light microscopy. The images were obtained at magnifications of 100× and 200× using the microscope. In order to determine the crypt depth (C) and villus height (V), light microscopic analysis was used in combination with digital measurement using Image J software (Bethesda, MD, USA). For mucin analysis, paraffin-embedded intestinal tissue sections were treated with AB-PAS staining.

### 2.4 Biochemical analysis

Key biomarkers-including aspartate aminotransferase (AST), alanine aminotransferase (ALT), glutathione peroxidase (GSH-Px), malondialdehyde (MDA), and superoxide dismutase (SOD) were analyzed in both serum and liver tissue samples. These measurements were conducted using standardized assay kits commercially available from Jiancheng Bioengineering Institute, located in Nanjing, China. Interleukin-6 (IL-6), tumor necrosis factor-α (TNF-α) and LPS plasma concentrations were measured with Solarbio ELISA kits (Beijing, China) per the manufacturer’s protocol.

### 2.5 Western blot assay

To evaluate protein expression levels of TRPV4, TLR4, key MAPK pathway elements (including JNK, p-JNK, ERK, p-ERK, p38, and p-p38), as well as tight junction proteins occludin and ZO-1, were performed Western blot analysis on small intestine and liver samples. Tissue homogenates were prepared from both the small intestinal and hepatic specimens prior to examination. The supernatant containing proteins was then collected and stored. The protein levels were evaluated using a BCA assay kit (Beyotime, Shanghai, China). Proteins were separated by 10% SDS-PAGE, followed by transfer onto PVDF membranes, which were then incubated with 5% BSA for 120 min to block non-specific binding. Following the initial step, the membranes were incubated with the primary antibodies at 4 °C overnight. To ensure proper loading, we employed the GAPDH protein as an internal control. Then the membranes were incubated with the appropriate HRP-conjugated secondary antibodies. The bands were visualized with an chemiluminescence reagent (Tinon, China) and subsequently scanned and analyzed by Image J software (Bethesda, MD, USA).

### 2.6 Immunofluorescence staining

Immunofluorescence (IF) analysis was carried out following the typical staining protocols. Tissue samples were treated with 4% paraformaldehyde for 40 min, followed by permeabilization in 0.1% Triton X-100 for 20 min. Then, the samples were blocked with 10% normal goat serum in PBS followed by staining with occludin (1:200 dilution) or ZO-1 (1:200 dilution). The samples were visualized using a fluorescence microscope (Olympus) and measured using Image J software.

### 2.7 16S rRNA sequencing for intestinal flora analysis

Mouse cecal samples underwent 16S rRNA sequencing conducted by Lianchuan Biological Company in Hangzhou. Bacterial genomic DNA was extracted from cecal content using an Omega Stool DNA extraction kit (Omega Bio-Tech, GA), and subsequently amplified by PCR. The V3–V4 region of the 16S rRNA gene was amplified using conventional primers 341F (5′-CCTACGGGNGGCWGCAG-3′) and 805R (5′-GACTACHVGGGTATCTAATCC-3′). Afterward, following the instructions given by the manufacturer, the sequencing was performed using an Illumina HiSeq platform. Sequences with at least 97% similarity were grouped into the same Operational Taxonomic Unit (OTU) using the UCLUST algorithm in QIIME. The representative sequences from every OTU were used for the purposes of taxonomic recognition and phylogenetic examination. Alpha-diversity was assessed using the Chao1, ACE, Shannon, and Simpson indices. Principal-component analysis was used so as to visualize the separation between groups and recognize the variations in microbiome data among different classifications. The variances in microbial communities across groups were examined via Metastats analysis. Also, PICRUSt was utilized to yield predicted Kyoto Encyclopedia of Genes and Genomes (KEGG) annotations.

### 2.8 Determination of SCFAs by GC–MS

Gas chromatography/mass spectrometry (GC–MS) was used to determine SCFAs in mouse fecal content, including acetic acid, propionic acid, butyric acid, valeric acid, isobutyric acid, and isovaleric acid. SCFAs concentrations in mouse feces are reported in mmol/L. Established methods were followed for both sample pretreatment and reagent preparation (30, 31).

### 2.9 Statistical analysis

Means ± standard deviations (SD) were utilized to display results from three separate trials conducted in triplicate. The study’s statistical analysis was performed using Prism 9.0 (GraphPad Software, USA). The study utilized one-way ANOVA and t-tests, deeming *p*-values under 0.05 as statistically significant. Spearman’s correlation was used to analyze the relationships.

## 3 Results

### 3.1 Sinensetin ameliorates HFD-induced weight gain, hepatic steatosis, and liver injury

**Figure 1:**
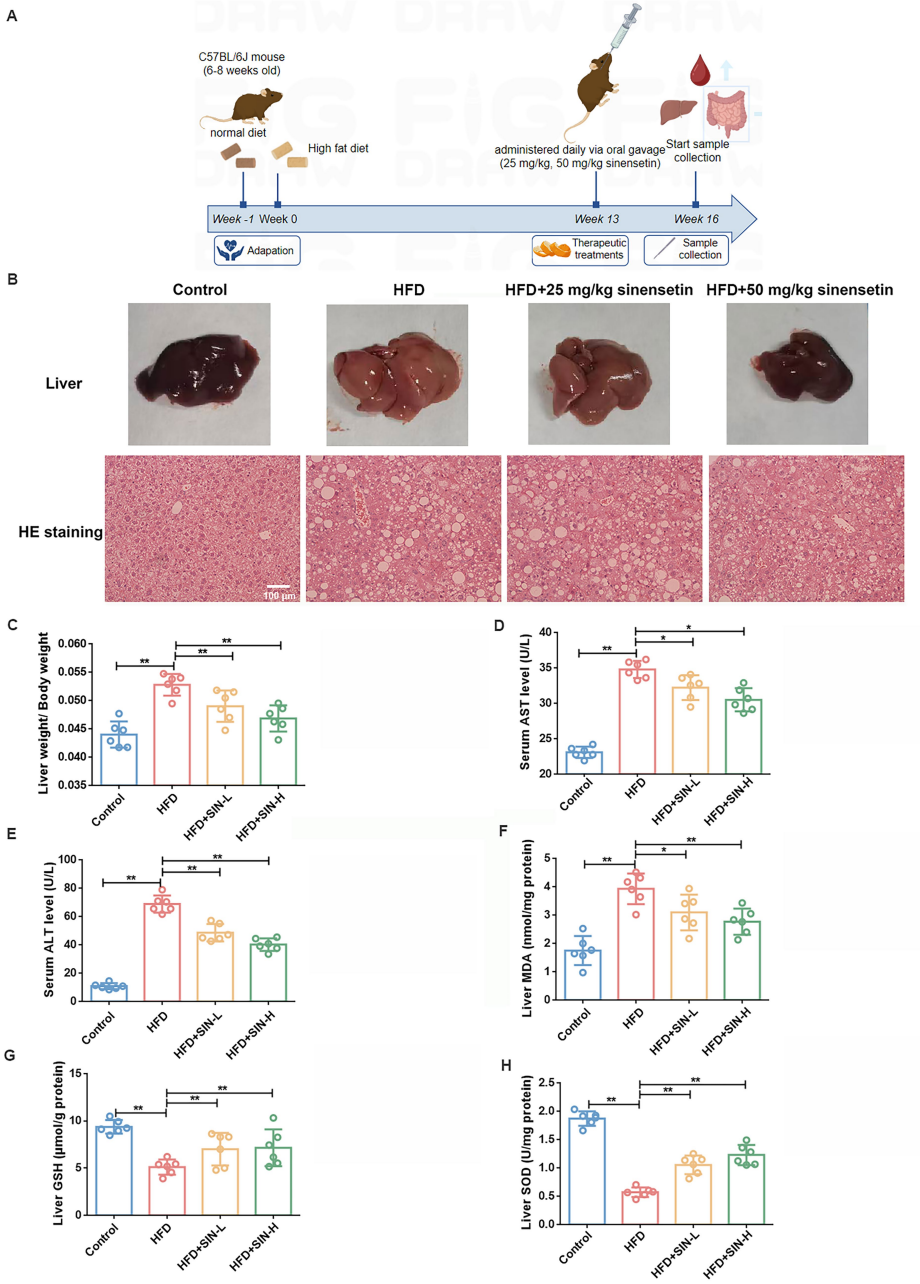
Sinensetin mitigated liver damage caused by an HFD in mice. **(A)** Animal experimental procedure (Drawn by Figdraw platform, ID: zzzzSc2512). **(B)** Morphological changes and H&E staining in mice. **(C–E)** The quantified parameters included serum concentrations of AST and ALT, along with the liver-to-body weight ratio. **(F–H)** The liver levels of MDA, GSH and SOD were detected. Average ± standard deviation, with a sample size of 6. Significance levels are denoted by ^*^p < 0.05 and ^**^p < 0.01, as shown in the brackets throughout the text.

A 16-week study on mice was conducted to assess sinensetin’s potential in reducing liver damage caused by an HFD. The grouping situation was depicted in Figure 1A. As shown in Figure 1B, the livers of HFD mice were notably larger and more yellow compared to the control group, including those administered with sinensetin. This suggested that the livers of HFD mice exhibited enhanced lipid accumulation. HE staining revealed that after 16 weeks of HFD feeding, regardless of sinensetin administration, the liver cells in control group mice maintained a well-structured organization. The liver cords were clear, and there was no significant detection of lipid deposits. In contrast, the hepatic cells in HFD-fed mice showed a chaotic arrangement. It was characterized by more obvious and severe steatosis, and there was also the presence of intralobular inflammatory foci and ballooning degeneration. The sinensetin groups exhibited reduced liver inflammation and steatosis compared to the HFD group. Compared with the control group, mice in the HFD group showed a notable rise in relative liver weight (1.2-fold change; *p* < 0.01), along with increased ALT (6-fold change; *p* < 0.01) and AST levels (1.4-fold change; *p* < 0.01). Compared with the HFD group, sinensetin intervention markedly reduced the concentration of the relative liver weight (1.2-fold change; *p* < 0.01), as well as serum ALT (1.8-fold change; *p* < 0.01) and AST (1.2-fold change; *p* < 0.05; Figures 1C–E). Evaluation of colon oxidative stress using MDA, GSH, and SOD assays showed that HFD-fed increased MDA (2.1-fold change; *p* < 0.01) but reduced GSH (1.9-fold change; *p* < 0.01) and SOD (3.5-fold change; *p* < 0.01) concentrations relative to the control group. Sinensetin substantially mitigated these detrimental outcomes, as illustrated in Figures 1F–H. Taken together, these results illustrated sinensetin’s ability to counteract the liver inflammation and damage typically caused by an HFD.

### 3.2 Sinensetin enhanced mitophagy, thereby inhibiting the TLR4/MAPK signaling pathway in HFD-fed mice

**Figure 2:**
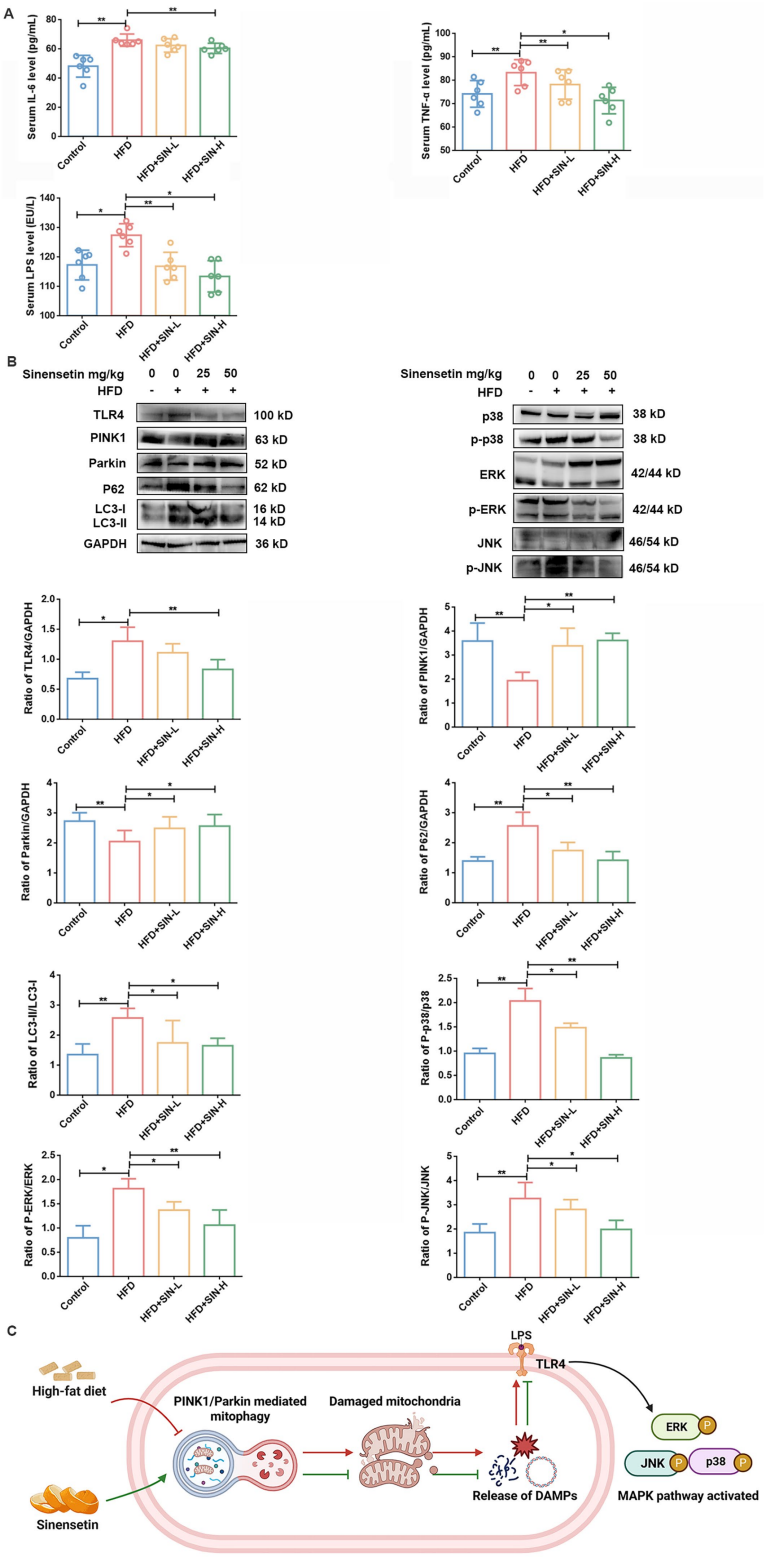
Sinensetin protected against HFD-induced liver damage by enhancing mitophagy, thereby inhibiting the TLR4/MAPK pathway. **(A)** Assessment of circulating LPS, IL-6 and TNF-α levels. **(B)** Relative protein expression of PINK1, Parkin, P62, LC3, TLR4, and p38, ERK, JNK in liver tissues. Average ± standard deviation, with a sample size of 3. **(C)** Pathway diagram. Significance levels are denoted by *p < 0.05 and **p < 0.01.

Consistent with our hypothesis, the HFD group exhibited significantly elevated levels of IL-6 (1.4-fold change; *p* < 0.01), TNF-α (1.1-fold change; *p* < 0.01) and LPS (1.1-fold change; *p* < 0.05) compared to the control group. In contrast, sinensetin treatment effectively reduced these abnormal elevations of inflammatory cytokines (Figure 2A). The molecular mechanism by which sinensetin restored HFD-suppressed hepatic mitophagy and subsequently inhibited MAPK signaling was further investigated. Western blot analysis provided evidence that sinensetin remarkably reduced the phosphorylation levels of p38 (2.5-fold change; *p* < 0.01), ERK (1.7-fold change; *p* < 0.05), JNK (1.5-fold change; *p* < 0.01), and the levels of TLR4 (1.6-fold change; *p* < 0.05), P62 (1.8-fold change; *p* < 0.01) and LC3 (1.5-fold change; *p* < 0.01), induced the levels of PINK1 (1.8-fold change; *p* < 0.01) and Parkin (1.3-fold change; *p* < 0.01; Figure 2B). Therefore, by modifying the mitophagy/TLR4/MAPK signaling, sinensetin may reduce HFD-stimulated liver injury (Figure 2C).

### 3.3 Sinensetin administration improved the histological structure of the small intestine

**Figure 3:**
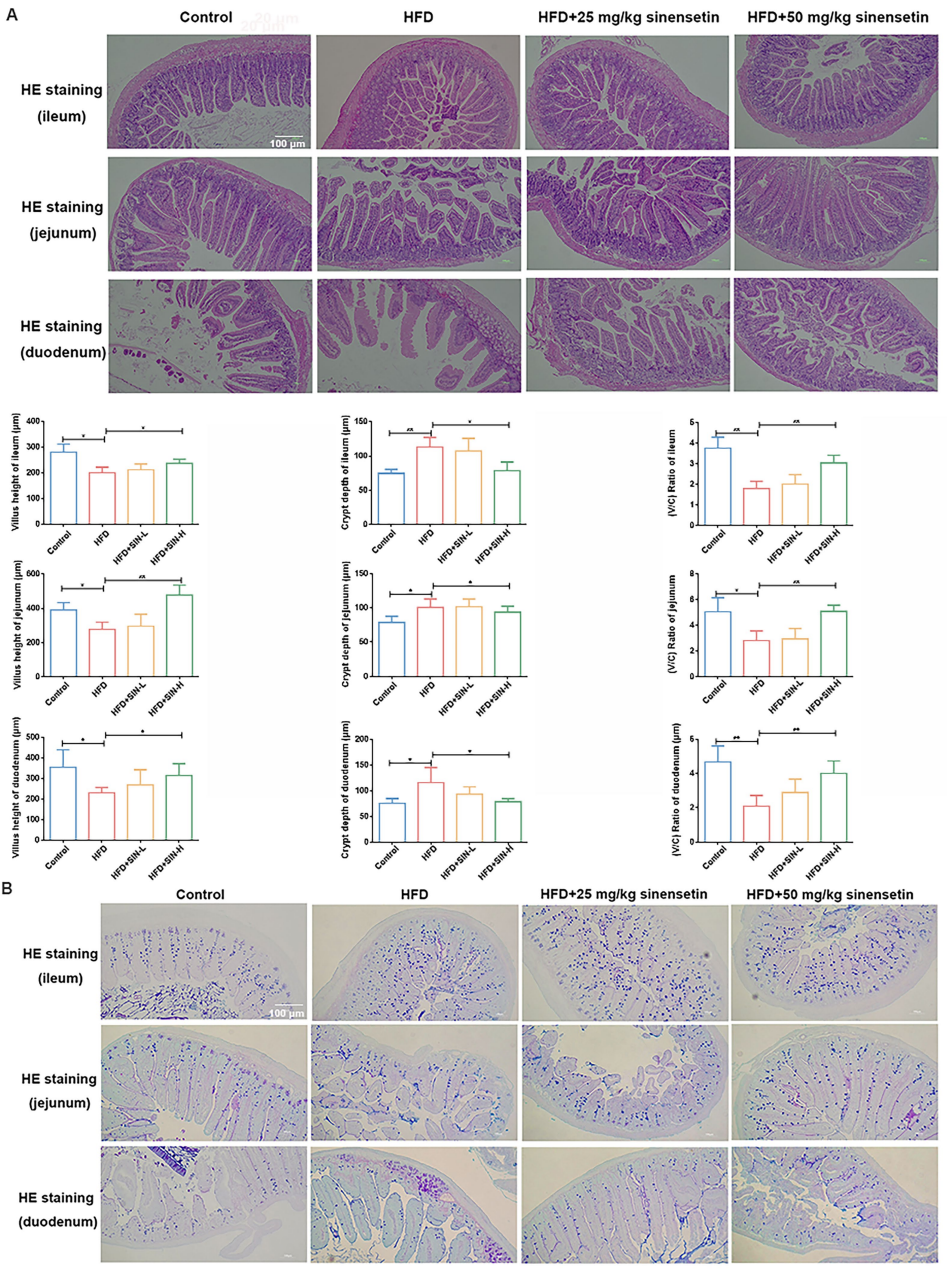
Sinensetin ameliorated intestine damage induced by HFD in mice. **(A)** H&E staining (scale bar 100 μm) was used to assess the villus height, crypt depth, and the villus height to crypt depth ratio (V/C) in the ileum, jejunum, and duodenum. **(B)** AB-PAS staining of ileum, jejunum, and duodenum (scale bar: 100 μm). Significance levels are denoted by *p < 0.05 and **p < 0.01.

The histopathological assessment of the small intestine was presented in Figure 3A. Tissues from the normal group demonstrated structural integrity, with systematically arranged villi, deep crypts, and clearly visible brush borders. The administration of an HFD notably disrupted the architecture and arrangement of intestinal villi. A notable decrease in villus height and the V/C ratio across the duodenum, jejunum, and ileum were observed alongside an increase in crypt depth in these areas. Notably, sinensetin application mitigated HFD-induced intestinal morphological disruptions. This led to an increase in villi height and the V/C ratio, while simultaneously decreasing crypt depth. The AB-PAS staining results depicted in Figure 3B showed that sinensetin counteracted the HFD-induced decrease in goblet cell count and maintained mucosal barrier integrity in the duodenum, jejunum, and ileum. The findings demonstrate that sinensetin administration exerts a restorative effect on small intestinal morphology.

### 3.4 Effect of sinensetin on intestinal barrier disruption in HFD-fed mice

**Figure 4:**
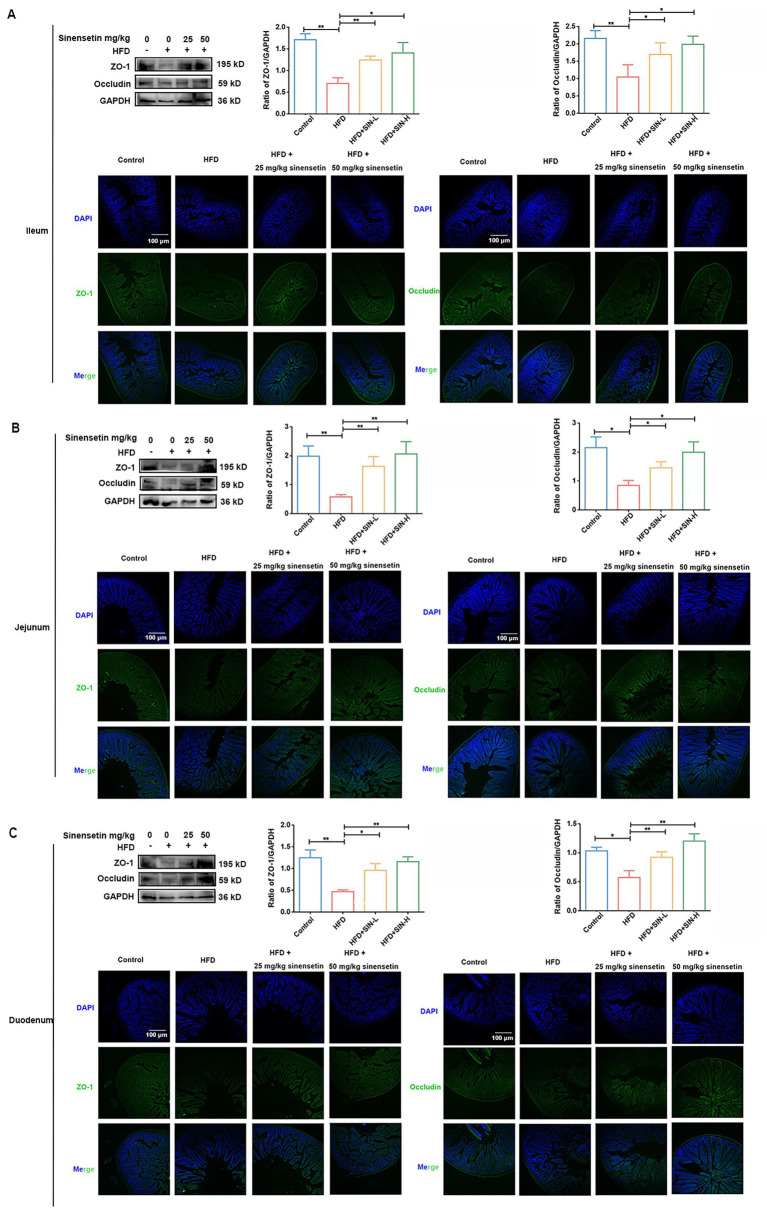
Impact of sinensetin on intestinal barrier integrity and permeability in mice fed an HFD. **(A)** Immunofluorescence analysis and relative protein expression of ZO-1 and occludin in the ileal tissue of mice. **(B)** Immunofluorescence analysis and relative protein expression of ZO-1 and occludin in jejunal segments. **(C)** Immunofluorescence analysis and relative protein expression of ZO-1 and occludin in duodenal tissues (scale bar: 10 μm). Significance levels are denoted by *p < 0.05 and **p < 0.01.

ZO-1 and occludin are crucial components of tight junctions, essential for preserving barrier function (32). We investigated the impact of sinensetin on the expression of tight junction proteins ZO-1 and occludin in the duodenum, jejunum, and ileum using Western blot analysis. Sinensetin treatment effectively restored the protein levels of ZO-1 and occludin, which were downregulated in the model group (Figure 4). Immunofluorescence analysis in the control group demonstrated a distinct localization of ZO-1 and occludin in the intestinal epithelium of the duodenum, jejunum, and ileum. HFD consumption significantly reduced the fluorescence intensity of ZO-1 and occludin. Conversely, sinensetin exerted a dual benefit by both augmenting the fluorescence intensity of these proteins and promoting the restoration of intestinal structural integrity. The findings indicated that sinensetin provided protection against intestinal barrier dysfunction induced by an HFD in mice.

### 3.5 The composition of microbial communities was modified by sinensetin

**Figure 5:**
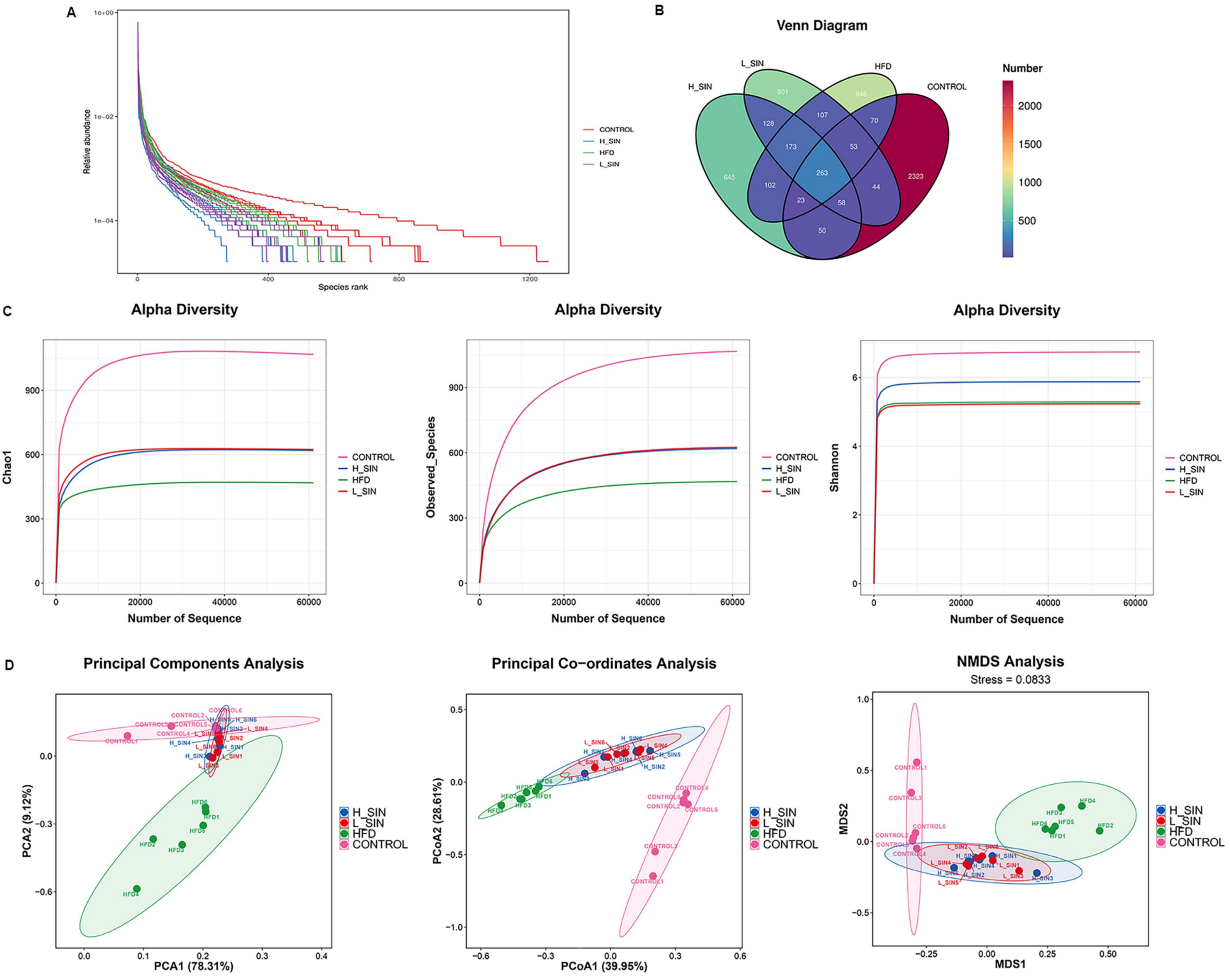
Impacts of sinensetin treatment on the gut microbiome in mice subjected to an HFD. **(A)** Recorded OUTS. **(B)** Analysis of shared and unique OTUs via Venn diagram. **(C)** Alpha diversity metrics (Chao1, Shannon, and ACE indices). **(D)** PCA of OTU abundance and PCoA of weighted UniFrac distances and NMDS analysis based on Bray-Curtis distance.

Assessment of the intestinal microbiome following sinensetin treatment was conducted via 16S rRNA gene sequencing. The results illustrated in Figure 5A indicated that the curve for the normal group was highest along the X-axis, implying the greatest abundance of intestinal microbiota species in the normal control group. Conversely, the HFD group’s curve was the lowest along the X-axis, suggesting a decrease in species abundance. The sinensetin groups closely resembled the normal group. The depiction of OTUs via a Venn diagram showed a core microbiome shared between groups alongside treatment-specific OTUs, highlighting how the interventions modulated community composition (Figure 5B). Figure 5C illustrated that the Chao1 and Shannon rarefaction curves have reached a plateau, suggesting that the sampling of tags for sequencing was both sufficient and appropriate. Clear segregation of the four groups was observed in the Principal coordinates analysis (PCoA) analysis using Bray-Curtis metrics (Figure 5D), indicating significant inter-group divergence in gut flora. The stress value of 0.0833, which was below the 0.1 threshold, indicated effective dimensionality reduction and accurate data structure representation. PCoA utilized weighted UniFrac distances for evaluation. Bray-Curtis distances were analyzed via non-metric multidimensional scaling (NMDS). The Shannon index was evaluated through the Tukey-HSD test, while the Bray–Curtis distance underwent analysis with the Kruskal–Wallis *H* test.

**Figure 6:**
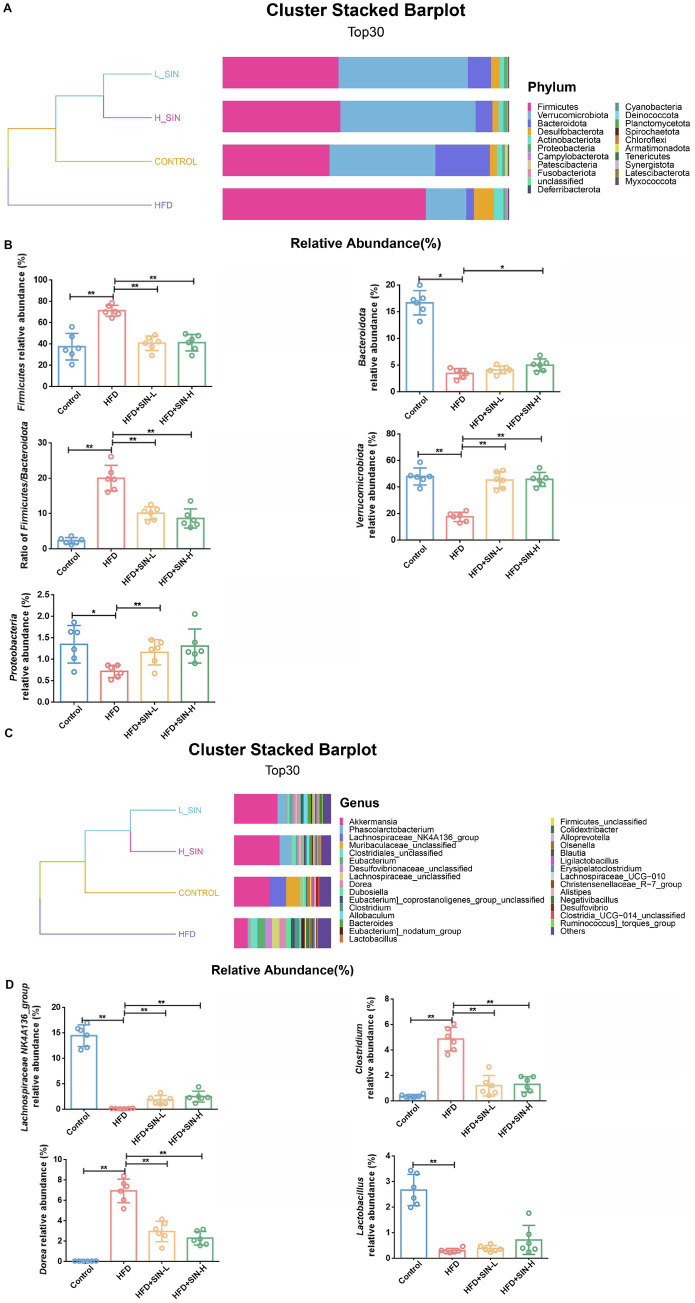
The application of sinensetin has an impact on the constitution of the intestinal microbiota in mice subjected to an HFD. **(A)** This panel displays the microbial community breakdown across different phyla. **(B)** Relative proportions of gut microbiota at the phylum level. **(C)** Genus-based breakdown of intestinal microbiota composition. **(D)** Relative abundance of gut microbial taxa at the genus level. Significance levels are denoted by *p < 0.05 and **p < 0.01.

**Figure 7:**
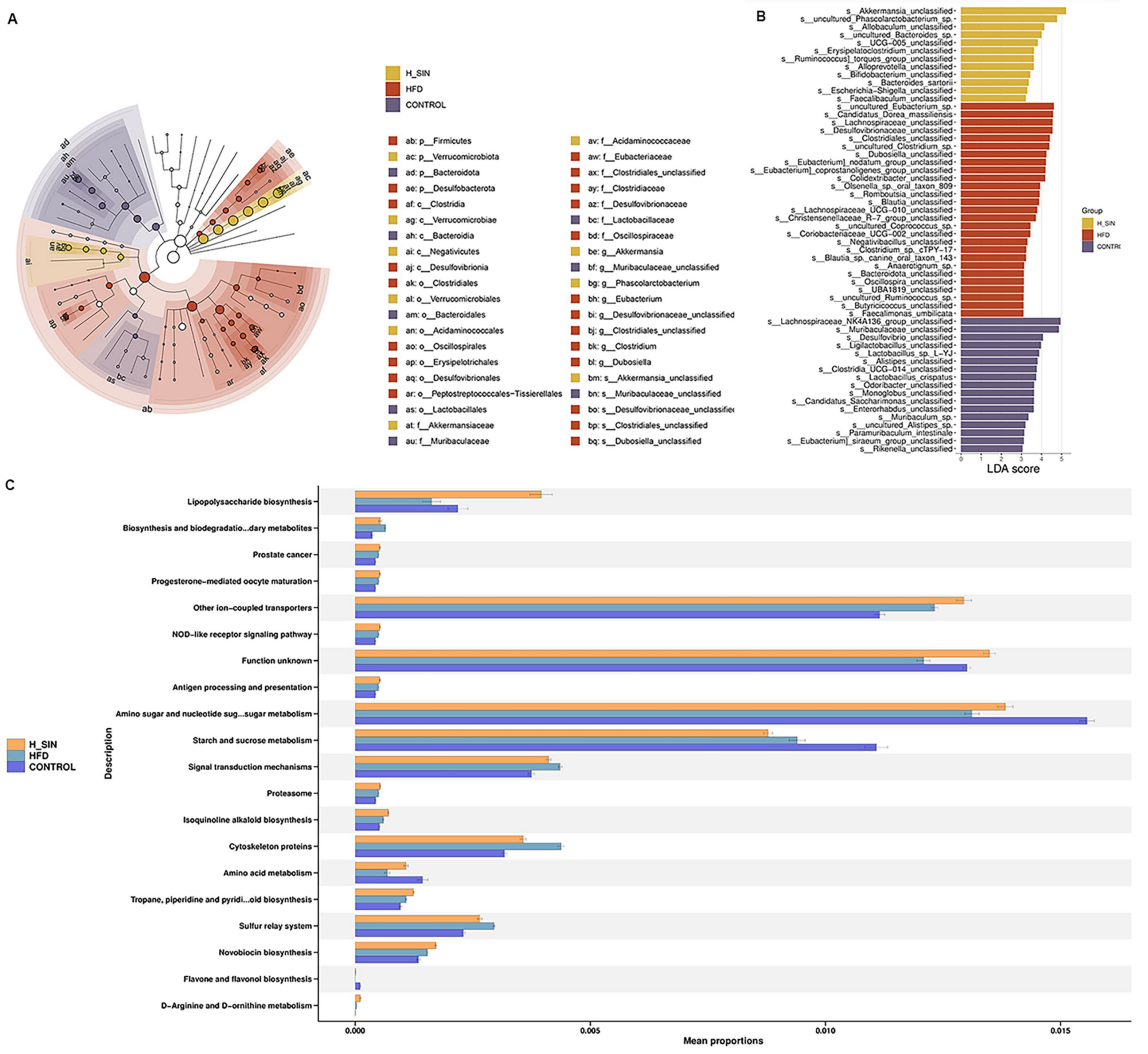
LEfSe analysis and prediction of pathways for differential gut microbiota utilizing PICRUSt analysis. **(A)** Taxonomic cladogram generated by LEfSe. **(B)** A bar chart depicting the LEfSe scores along with the relative abundance of significantly altered bacterial species among the three groups **(C)** Pathways that showed differences between the control group, the HFD group, and sinensetin group as recognized by the KEGG database.

The analysis of intestinal microbial composition within cecal contents across different groups was conducted. Figure 6A illustrated the relative abundance of microbial communities at the phylum level, highlighting 10 dominant phyla in the cecal microbiota: *Firmicutes*, *Verrucomicrobiota*, *Bacteroidota*, *Desulfobacterota*, *Actinobacteriota*, *Proteobacteria*, *Campilobacterota*, *Patescibacteria*, *Fusobacteriota*, and *Deferribacterota*. At the phylum level, all four groups were dominated by *Firmicutes* and *Bacteroidetes*. Figure 6B demonstrated that HFD treatment notably increased the *Firmicutes* to *Bacteroidetes* (F/B) ratio (7-fold change; *p* < 0.01). Sinensetin treatment enhanced the relative abundances of *Verrucomicrobiota* (2-fold change; *p* < 0.01) and *Patescibacteria* (1.5-fold change; *p* < 0.05) compared to the HFD group. The species-level richness of the intestinal microbial community was further analyzed. Figure 6C revealed that the gut microbiota of mice predominantly comprised 10 species: *Akkermansia*, *Phascolarctobacterium*, *Lachnospiraceae_NK4A136_group*, *Eubacterium*, *Dorea*, *Dubosiella*, *Clostridium*, *Allobaculum*, *Bacteroides*, and *Eubacterium_nodatum_group*. Sinensetin administration increased the relative abundance of *Lachnospiraceae_NK4A136_group* (11-fold change; *p* < 0.01) and *Lactobacillus* (1.3-fold change; *p* > 0.05) compared to the HFD group. The HFD group showed a higher relative abundance of *Dorea* and *Clostridium*, while sinensetin administration reduced the abundance of these genera. We applied a feature selection criterion requiring a linear discriminant analysis (LDA) score above 3 to perform a linear discriminant analysis effect size (LEfSe) analysis, identifying biomarkers linked to the bacterial communities in both groups. The taxonomic cladogram (Figure 7A) and the histogram illustrating species distribution based on the LDA value (Figure 7B) provided deeper insights. To elucidate the functional shifts within the gut microbiome, PICRUSt2 was utilized to predict KEGG pathways. The analysis revealed that the HFD group significantly upregulated pathways linked to LPS biosynthesis and NOD-like receptor signaling, while disrupting ion-coupled transporters. The enrichment of LPS biosynthesis provided a direct microbial basis for HFD-induced endotoxemia, where microbiota-derived LPS crosses the compromised gut barrier to activate the hepatic TLR4/MAPK inflammatory cascade. Concurrently, upregulated NOD-like receptor signaling reflects exacerbated intracellular immune responses and oxidative stress. Crucially, sinensetin intervention effectively reversed these pathological shifts. It markedly suppressed the LPS-synthetic and NOD-like receptor pro-inflammatory pathways, while concurrently enriching metabolic modules responsible for SCFA synthesis. These functional predictions corroborated our *in vivo* findings, demonstrated that sinensetin mitigated liver injury by reprogramming the gut microbiome towards a barrier-protective, SCFA-producing, and anti-inflammatory phenotype.

### 3.6 Sinensetin counteracted the suppression of SCFAs production caused by an HFD

**Figure 8:**
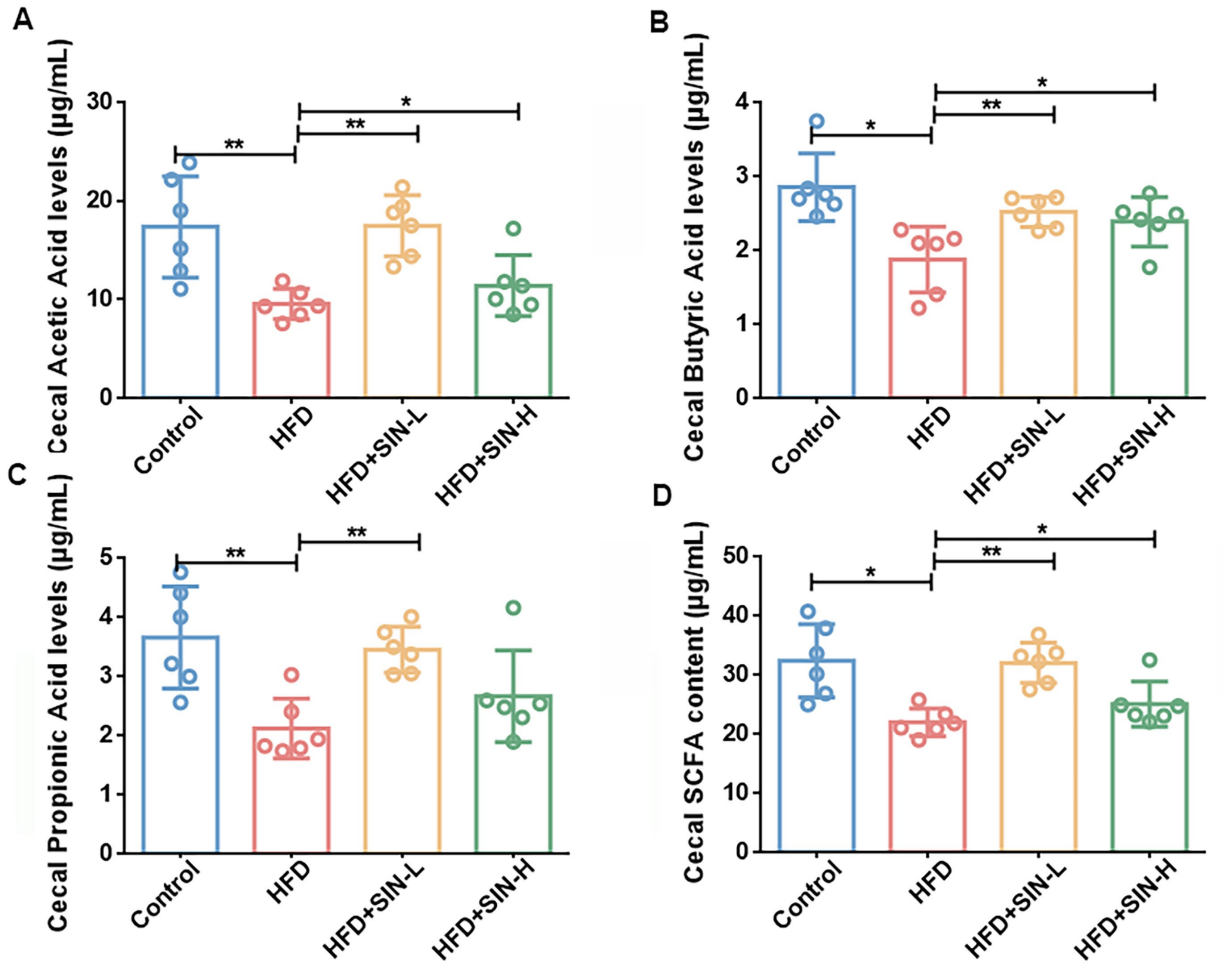
Determination of SCFA content. **(A)** Contents of acetic acid. **(B)** Contents of butyric acid. **(C)** Contents of propionic acid. **(D)** Contents of total SCFAs. Significance levels are denoted by *p < 0.05 and **p < 0.01.

The gut’s microflora produces small-chain fatty acids, mainly acetate, propionate, and butyrate. Research indicates that an increase in the levels of these SCFAs can strengthen the gut’s protective barrier (33). Notably, the HFD group exhibited a significant decrease in total SCFAs, in stark contrast to the sinensetin groups. In addition, the contents of acetic acid (1.9-fold change; *p* < 0.05), propionic acid (1.3-fold change; *p* < 0.01), and butyric acid (1.3-fold change; *p* < 0.05) were significantly reduced in the HFD group, while they were significantly increased after treatment with sinensetin (Figure 8). This suggested that sinensetin significantly influenced the regulation of specific SCFAs, metabolited produced by gut microbiota.

### 3.7 Correlation analysis

**Figure 9:**
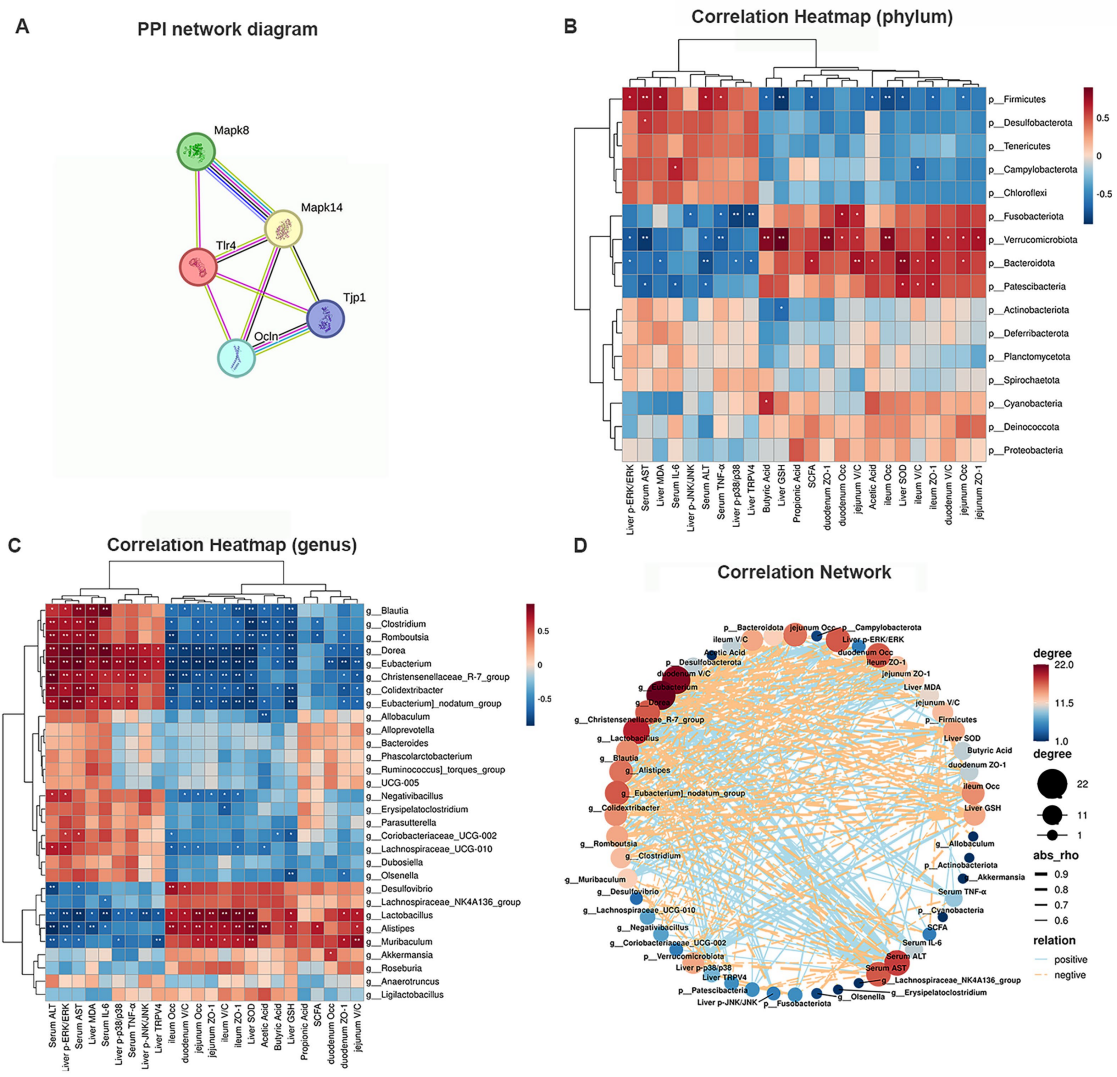
Correlation analysis. **(A)** PPI network illustrating key molecular relationships. **(B)** Heatmap depicting the phylum-level associations between gut microbiota composition and hepatic injury. **(C)** Genus-level heatmap analysis examining the interplay between gut microbial communities and liver dysfunction. **(D)** Network analysis highlighting the correlation between critical hepatic markers and gut microbiota indicators.

The study utilized the STRING database to analyze the protein–protein interaction (PPI) network. It explored the associations between gut microbiota, SCFAs, the TLR4/MAPK signaling pathway, liver inflammation markers (such as TNF-α and IL-6), and intestinal permeability indicators (for example, ZO-1 and Occludin), as depicted in Figure 9A. The total number of nodes was 6 with 13 edges (the expected number of edges was 4). The *p*-value for PPI analysis was determined to be 0.000134, suggesting strong protein interactions. In addition, to further investigate the relationship between gut microbiota, intestinal permeability, and liver damage, a Spearman correlation analysis was conducted (Figures 9B–D). At the genus level, significant positive correlations were observed between the abundance of *g__Alistipes*, *g__Bacteroides*, *g__Alloprevotella*, *g__Akkermansia*, and *g__Lachnospiraceae_NK4A136_group* and the production of SCFAs. A significant negative correlation was observed between the abundance of *g__Romboutsia*, *g__Clostridium*, *g__Colidextribacter*, *g__Dubosiella*, *g__Coriobacteriaceae_UCG-002*, and *g__Ruminococcus_torques_group* and SCFA production. Furthermore, liver inflammation was negatively correlated with elevated levels of *g__Lactobacillus*, *g__Lachnospiraceae_NK4A136_group*, *g__Muribaculum*, and *g__Roseburia*. The results indicated that gut microbiota may significantly contribute to intestinal damage and liver injury associated with a HFD. SCFAs positively correlated with MDA, SOD, and GSH levels, and negatively correlated with TLR4/MAPK pathway proteins. In summary, sinensetin may contribute to ameliorating gut microbiota imbalance and reducing liver damage induced by an HFD via the gut–liver axis.

## 4 Discussion

In this study, we established a mouse model of liver injury induced by an HFD and revealed through molecular experiments that sinensetin pretreatment may contribute to protecting mice from liver injury by orchestrating the integrated microbiota-SCFA-mitophagy network, which subsequently suppressed the downstream TLR4/MAPK signaling pathway, and this protective effect was achieved through the gut–liver axis.

HFD-induced liver damage involves various molecular pathways, including the activation of TLR4 and MAPK signaling cascades (34, 35). The buildup of lipids and the presence of endotoxins lead to an increase in TLR4, a receptor that plays a key role in identifying molecular patterns. This heightened activation triggers the NF-κB pathway, which in turn induces the secretion of inflammatory signaling molecules such as TNF-α, IL-1β, and IL-6 (34, 35). ROS-induced activation of these MAPK pathways enhances PINK1 stability and its interaction with Parkin (36). The interplay between mitophagy and TLR4/MAPK signaling pathways highlights the regulatory mechanisms in HFD-induced liver injury, suggesting potential therapeutic targets within these pathways to mitigate liver damage. Our findings proposed that sinensetin not only ameliorated downstream hepatic steatosis and local inflammatory responses, but also exerted their protective effects by preemptively neutralizing inflammatory reactions at the upstream source of the inflammatory cascade. Ultimately, it acted through restoration of PINK1/Parkin-dependent mitophagy and subsequent inhibition of the TLR4/MAPK signaling axis. Our findings align with other studies that have documented the beneficial effects of sinensetin. A previous study indicated that sinensetin might improve NAFLD induced by an HFD (25). Moreover, the research has indicated that sinensetin can inhibit NF-κB and MAPK signaling pathways (37). The current study’s results were consistent with those found by Javedan et al. and Ajami et al., where it was demonstrated that CLA and DHA + EPA treatments modulate oxidative stress and apoptosis in ischemia/reperfusion induced renal injury (4, 5). While both of these studies implicated the mTOR pathway, the present work extended these findings by indicating sinensetin’s cytoprotective effects occured via mitophagy and the regulation of TLR4/MAPK pathways.

A symbiotic connection exists between the liver, the gastrointestinal tract, and the gut microbiome. External agents can alter microbiota composition, promoting harmful bacteria growth while reducing beneficial bacteria. This change commonly results in gut barrier dysfunction and microbial transfer to the liver. Extensive research has consistently shown that diets rich in fat disrupt the balance of gut microbiota, prompting investigations into how these dietary patterns contribute to concurrent liver dysfunction and intestinal harm (38, 39). This study also examined whether sinensetin could mitigate diet-induced hepatic damage. The study focused on enhancing the gut barrier’s structural integrity and its effect on the gut’s microbial community via the gut–liver interaction. The structural degradation of the intestinal mucosa and the loss of goblet cells observed in HFD-fed mice underscored that hepatic injury was not an isolated event but a downstream consequence of gut barrier failure. The TJ structure, a key component of a dynamic protein network, is integral to the intestinal epithelial cells, as it plays a vital role in maintaining the integrity of the intestinal barrier (40). Furthermore, it is considered essential for preserving optimal barrier function (41). Immunofluorescence analysis confirmed that sinensetin restored the expression of ZO-1 and occludin, which were downregulated by an HFD. This reinforced the intestinal mucosal barrier, blocked LPS translocation at its source, and provided an upstream prophylactic strategy against hepatic lesions.

The gut microbiome is crucial for maintaining intestinal micro-environment stability, supporting intestinal barrier functions, and modulating immune responses (42). An HFD disrupts the gut microbiome, leading to increased intestinal permeability. This disruption enables bacterial endotoxins like LPS to enter the portal bloodstream (43). LPS activates TLR4. Through receptor binding on Kupffer cells and hepatocytes, it induces pro-inflammatory cytokine release, exacerbating hepatic injury (34, 35). This study observed changes in gut microbial composition induced by sinensetin. *Firmicutes* and *Bacteroides* are two dominant phyla in the gut microbiota. Previous research indicates that adverse shifts in the intestinal F/B ratio contribute to the dysregulation of lipid metabolism (44). In this study, sinensetin reduced the F/B ratio to levels similar to the control group. Furthermore, it successfully normalized the HFD-induced abnormal expansion of specific SCFA-producing taxa, including *g__Alistipes*, *g__Bacteroides*, *g__Alloprevotella*, *g__Akkermansia*, and *g__Lachnospiraceae_NK4A136_group*. SCFAs account for about 5–10% of the energy reserves in the host (45). The restoration of SCFA profiles following sinensetin treatment represented a key mechanism underlying its protective effects. Notably, our study revealed a novel integrative axis in which microbiota-derived SCFAs act as upstream regulators that activate PINK1/Parkin-mediated mitophagy. By clearing damaged mitochondria and reducing oxidative stress, this mitophagy effectively suppresses the downstream TLR4/MAPK inflammatory cascade. The HFD group exhibited a distinct microbiota composition, characterized by the presence of *Lachnospiraceae_NK4A136_group*, *Dorea*, and *Clostridium*, in contrast to the control group. Research indicates that a 16-week oral resveratrol regimen notably elevated the *Lachnospiraceae_NK4A136_group*, enhancing insulin sensitivity and lipid metabolism in HFD-fed mice (46). In addition, *Dorea* and *Clostridium* are known SCFA producers (47). Following treatment with sinensetin, there was a notable decrease in the relative abundances of the *Lachnospiraceae_NK4A136_group*, suggesting its role in mitigating the progression of liver injury induced by an HFD. This study suggested that gut microbiota instability may worsen liver injury caused by an HFD. In addition, SCFAs presented a positive association with the concentrations of SOD and GSH. At the same time, they exhibited a strong positive correlation with mitophagy-activating proteins and an inverse correlation with the pro-inflammatory TLR4/MAPK pathway. Moreover, corroborating evidence from various studies aligns with our findings that suppressing the TLR4/NF-κB/MAPK signaling pathway averts HFD-induced liver damage (48, 49). Despite these promising mechanistic insights, several limitations of the present study must be acknowledged, including the relatively small sample size (*n* = 6) in the animal experiments. Although this was sufficient to detect statistically significant trends in acute pathology, larger cohorts are necessary to validate the long-term impacts of sinensetin on microbiome remodeling. Additionally, the absence of a baseline cohort at week 12 prevents definitive conclusions regarding whether the 4-week sinensetin intervention actively reversed pre-existing hepatic lesions or merely halted their progression relative to the 16-week HFD group; thus, staged intermediate assessments are needed to distinguish reparative from disease-arresting effects. Finally, future studies using gene knockout models (e.g., TLR4−/− mice) and comprehensive metabolomics are warranted to definitively trace the biotransformation and specific target binding sites of sinensetin in the gut.

Collectively, these results indicated that sinensetin had the potential to mitigate HFD-induced liver injury through integrating gut microbiota remodeling, SCFA enrichment, and mitophagy activation. This protective mechanism functions through mitophagy and the gut–liver axis. Our research will provide a relative reference on the theoretical understanding and commercial applicability of sinensetin in addressing food safety concerns related to HFD in food formulations and healthcare sectors.

## Data Availability

The original contributions presented in the study are included in the article/supplementary material, further inquiries can be directed to the corresponding authors.
